# InterVA-4 as a public health tool for measuring HIV/AIDS mortality: a validation study from five African countries

**DOI:** 10.3402/gha.v6i0.22448

**Published:** 2013-10-18

**Authors:** Peter Byass, Clara Calvert, Jessica Miiro-Nakiyingi, Tom Lutalo, Denna Michael, Amelia Crampin, Simon Gregson, Albert Takaruza, Laura Robertson, Kobus Herbst, Jim Todd, Basia Zaba

**Affiliations:** 1 WHO Collaborating Centre for Verbal Autopsy, Umeå Centre for Global Health Research, Umeå University, Umeå, Sweden; 2 School of Public Health, University of the Witwatersrand, Johannesburg, South Africa; 3 INDEPTH Network, Accra, Ghana; 4 London School of Hygiene and Tropical Medicine, London, UK; 5 MRC/UVRI Uganda Research Unit on AIDS, Entebbe, Uganda; 6 Rakai Health Sciences Program, Rakai, Uganda; 7 National Institute for Medical Research, Dar es Salaam, Tanzania; 8 Karonga Prevention Study, Karonga, Malawi; 9 Manicaland HIV-STD Prevention Project, Zimbabwe; 10 Imperial College School of Public Health, London, UK; 11 Africa Centre for Health and Population Studies, University of KwaZulu-Natal, KwaZulu-Natal, South Africa

**Keywords:** HIV/AIDS, mortality, Africa, verbal autopsy, InterVA, Alpha Network

## Abstract

**Background:**

Reliable population-based data on HIV infection and AIDS mortality in sub-Saharan Africa are scanty, even though that is the region where most of the world’s AIDS deaths occur. There is therefore a great need for reliable and valid public health tools for assessing AIDS mortality.

**Objective:**

The aim of this article is to validate the InterVA-4 verbal autopsy (VA) interpretative model within African populations where HIV sero-status is recorded on a prospective basis, and examine the distribution of cause-specific mortality among HIV-positive and HIV-negative people.

**Design:**

Data from six sites of the Alpha Network, including HIV sero-status and VA interviews, were pooled. VA data according to the 2012 WHO format were extracted, and processed using the InterVA-4 model into likely causes of death. The model was blinded to the sero-status data. Cases with known pre-mortem HIV infection status were used to determine the specificity with which InterVA-4 could attribute HIV/AIDS as a cause of death. Cause-specific mortality fractions by HIV infection status were calculated, and a person-time model was built to analyse adjusted cause-specific mortality rate ratios.

**Results:**

The InterVA-4 model identified HIV/AIDS-related deaths with a specificity of 90.1% (95% CI 88.7–91.4%). Overall sensitivity could not be calculated, because HIV-positive people die from a range of causes. In a person-time model including 1,739 deaths in 1,161,688 HIV-negative person-years observed and 2,890 deaths in 75,110 HIV-positive person-years observed, the mortality ratio HIV-positive:negative was 29.0 (95% CI 27.1–31.0), after adjustment for age, sex, and study site. Cause-specific HIV-positive:negative mortality ratios for acute respiratory infections, HIV/AIDS-related deaths, meningitis, tuberculosis, and malnutrition were higher than the all-cause ratio; all causes had HIV-positive:negative mortality ratios significantly higher than unity.

**Conclusions:**

These results were generally consistent with relatively small post-mortem and hospital-based diagnosis studies in the literature. The high specificity in cause of death attribution achieved in relation to HIV status, and large differences between specific causes by HIV status, show that InterVA-4 is an effective and valid tool for assessing HIV-related mortality.

As the HIV/AIDS pandemic has evolved, reliable assignment of cause of death for people who have lived with the infection has been a persistent difficulty. Although there is no doubt about increased mortality risks associated with HIV infection (1), in settings where people frequently die outside the scope of effective medical services, or where the disease continues to carry some stigma, there may be inadequate information or unwillingness to document HIV/AIDS as a cause of death (2). The situation is further complicated by the interactions between HIV infection and other diseases, whereby clear manifestations of AIDS may not be major features of final illnesses among HIV-positive people, particularly among those receiving treatment (3).

As a result of these difficulties, reliable community-based data on HIV/AIDS-related mortality, particularly in highly HIV-infected areas in Africa, have been very scanty. Modelled estimates of AIDS mortality at national and global levels have therefore fluctuated widely as the pandemic has progressed, and despite improving estimation methods, details remain uncertain (4). It is widely recognised that where deaths are not routinely certified as to their cause, verbal autopsy (VA) is the interim method of choice for documenting cause-specific mortality patterns (5). Although VA has been variously implemented in areas of high HIV infection and AIDS mortality, VA procedures have generally not been validated at the individual level against measurable outcomes such as HIV sero-status, leading to suppositions that VA is an unreliable approach for identifying HIV/AIDS deaths (6). Some sero-validation studies have been implemented in the context of HIV treatment centres (7), but not on a population basis, where there is normally no information on pre-mortem infection status. Other studies have compared VA results on AIDS mortality from different sources, such as between InterVA and physician interpretation, but in the absence of any absolute standard for comparison (8).

In a collaboration between the Health Metrics Network (9), the Alpha Network (10), and the INDEPTH Network (11), one aim was to validate measurement of AIDS mortality in documented population surveillance sites in which HIV infection status was gathered prospectively. This initiative coincided with the development of the InterVA-4 model for interpreting VA interview material, the latest model in the InterVA series (12) which corresponds to the 2012 WHO VA tool (13) and is freely available at www.interva.net.

The aim of this article is to analyse InterVA-4 interpretations of routinely gathered VA material from six population surveillance sites in Africa, as shown in Fig. 1, against known HIV sero-status. This will permit an assessment of the specificity of the InterVA-4 model for the assignment of HIV/AIDS-related deaths, as well as investigating differences in cause of death patterns according to HIV infection as a risk factor for mortality in African populations.

**Fig. 1:**
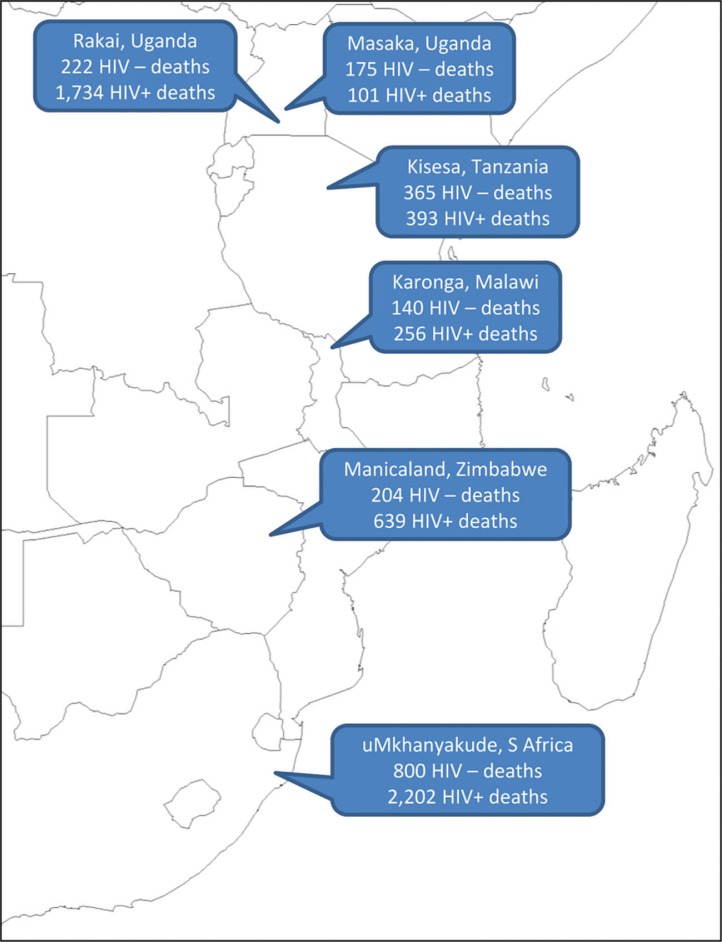
Participating Alpha Network sites, showing numbers of deaths with verbal autopsies, by HIV status.

## Methods

The basic modes of operation of the six population surveillance sites involved, of which all are members of the Alpha Network and some are members of the INDEPTH Network, have been described previously (14–19). These sites are located on a north–south transect of some 3,500 km through eastern and southern Africa. Briefly, all the sites followed a particular geographically defined adult population longitudinally, capturing vital events as well as the results of HIV sero-testing. Exact procedures for following up deaths and carrying out VA interviews (interviews with relatives or other witnesses of a death about the circumstances and symptoms of the final illness) varied to some extent between sites, and have developed over time. Intervals between deaths and VA follow-up vary across sites, ranging up to 2 years in Manicaland. For the purposes of this study, no special procedures were implemented, and it was important to make use of all available archived VA interview material, most of which was collected before current standards for undertaking VAs were established. Nevertheless, for the purposes of standard comparison over time and between sites, all the archived VA data were converted into the WHO 2012 standard VA format, which corresponds exactly to the input parameters needed for the InterVA-4 interpretative model (13). In some cases, desirable data items were not available in the VA archives, in which case they remained absent from the InterVA-4 input. However, the InterVA-4 model was deliberately designed to determine cause of death on the basis of available data items, and does not make any distinction between negative and missing values for individual parameters.

The data used for this validation study relate to 17,560 adult deaths (15 years and over) occurring between 1990 and 2011, all of which were successfully documented in VA interviews, from the six population sites shown in Fig. 1. Overall 5,029 (28.6%) of these cases had one or more documented HIV sero-test results sometime before death. Any recorded positive test was taken as evidence of HIV infection, and person-time from the test date onwards categorised as HIV positive. Recorded negative tests were only considered in relation to cause of death if documented within 5 years of death, and the time period between testing and death was recorded individually. HIV-negative person-time was calculated starting from any negative test result, and running until the first of: a positive test result, death, or the fifth anniversary of the negative test. In the majority of the VA interviews conducted, a specific question was included as to whether, to the knowledge of the respondent, the deceased had any history of HIV/AIDS diagnosis or treatment. If such a declaration was made in the VA interview for someone who had only documented a negative test, then those cases were put into a separate positive category, on the grounds that they had most likely sero-converted since testing. Similarly, a further positive category was defined for those who had not been sero-tested within 5 years of death, but were declared in VA interviews to have had a diagnosis of HIV. Specific questions about existing HIV diagnoses were not included in the VA instruments used in the Manicaland and Masaka sites. Those cases which had no sero-test recorded or whose most recent negative test result was recorded more than 5 years before death, and for which no diagnosis of HIV or AIDS was declared at VA, remained in an unknown status group. For the data input to the InterVA-4 model, if a diagnosis of HIV or AIDS was reported in the VA interview, then the corresponding input indicator was flagged, as would normally happen in the absence of serological data. In this study, the InterVA-4 model was not provided with any information on sero-test results, so that the cause of death assignment was blinded to those additional data.

Input indicators for the InterVA-4 model, as defined in the WHO 2012 VA tool, were retrospectively extracted from VA archives in each participating site. These were accumulated into a pooled dataset which was split into low malaria (South Africa) and high malaria (other sites) for InterVA-4 processing. This is in line with recommendations in the InterVA-4 documentation of high malaria corresponding to populations where approximately 1% or more of all deaths are due to malaria. Cause of death assignments (up to three per case) was made with associated likelihoods. Where input data are scanty, lower likelihood outputs typically result. As previously recommended with InterVA outputs, these data were analysed by taking the likelihoods of the assigned causes for each case and attributing any residual proportion of the death as indeterminate (12). InterVA-4 outputs were read into Stata 12 to process cause of death likelihoods. Consequently fractional causes of death arose, which have been aggregated in the following analyses. Cause of death categories assigned by InterVA-4 corresponds to the WHO 2012 VA standard cause of death groupings, one of which is ‘HIV/AIDS-related death’ (5). Specific causes which occurred very rarely were subsumed into the relevant residual categories.

Using data for individual residence episodes observed in the six participating sites, a Poisson regression model was constructed in Stata 12, using the sero-test results and cause of death findings to facilitate analysing cause-specific mortality fractions and rates by HIV infection status, allowing adjustment by age group, sex, and site.

## Results

Table 1 shows the total number of deaths, split by age group, sex, time period, and site, according to HIV infection status. Individuals who had reported negative tests are divided according to time period between testing and death, and positive individuals categorised according to the source of information, including recorded positive tests and diagnoses reported in VA interviews. Each cell shows the total number of deaths and the percentage of those assigned by InterVA-4 to ‘HIV/AIDS-related death’. It is not inevitable that all causes of death among HIV-positive people are HIV/AIDS related. Here, the overall proportion of deaths among the HIV positive attributed as HIV/AIDS-related was 41.4%. Deaths under the HIV-negative categories should, in principle, have no HIV/AIDS-related deaths assigned, and so those that are recorded must arise from sero-conversion after testing, false-positive assignments by the model, or errors in test records. These cases amounted to 9.9% of the HIV-negative deaths. The overall specificity with which InterVA-4 identified HIV/AIDS-related deaths was 90.1%. Table 2 shows the specificities by age group, sex, time period, and site.

**Table 1:** Numbers of adult deaths (*n=*17,560) and proportions assigned by InterVA-4 as HIV/AIDS related, by age, sex, time period, and site, according to HIV status

		HIV-negative test before death, at:				HIV-positive on the basis of:					
				
		0–11 months	12–23 months	24–59 months	Overall	Positive test and positive VA	Positive test, not VA	No test, positive VA	Prev. negative test, positive VA	Overall	HIV status unknown
Overall	Deaths	860	516	530	1,906	1,010	1,983	2,204	130	5,327	10,327
	% HIV/AIDS	8.4	9.4	12.8	9.9	45.8	36.7	43.4	43.0	41.4	15.4
15–49 years	Deaths	314	266	282	862	719	1,627	1,465	67	3,878	5,414
	% HIV/AIDS	16.1	13.9	20.1	16.7	51.2	39.3	49.0	55.1	45.4	23.8
50–64 years	Deaths	186	86	95	367	89	235	204	18	546	1,570
	% HIV/AIDS	6.8	7.5	8.2	7.3	37.4	32.6	45.4	48.1	38.7	12.8
65+ years	Deaths	360	164	153	677	202	121	535	45	903	3,343
	% HIV/AIDS	2.6	3.2	2.0	2.6	30.4	10.7	27.1	22.8	25.4	3.0
Male	Deaths	439	288	286	1,013	416	831	1,020	71	2,338	4,935
	% HIV/AIDS	5.1	7.3	6.4	6.1	39.9	27.9	38.0	39.9	34.8	12.4
Female	Deaths	421	228	244	893	594	1,152	1,184	59	2,989	5,392
	% HIV/AIDS	12.0	12.1	20.3	14.3	50.0	43.1	48.0	46.6	46.5	18.2
Pre-2002	Deaths	110	78	45	233	49	374	326	8	757	1,953
	% HIV/AIDS	24.6	18.1	26.5	22.8	70.1	58.8	53.0	85.1	57.3	20.6
2002–07	Deaths	299	200	253	752	545	1,173	1,199	59	2,976	6,338
	% HIV/AIDS	10.9	12.4	15.7	12.9	51.6	36.1	47.4	55.0	43.9	15.7
2008 on	Deaths	451	238	232	921	416	436	679	63	1,594	2,036
	% HIV/AIDS	2.9	4.1	6.9	4.2	35.5	19.5	31.7	26.3	29.1	9.3
Karonga	Deaths	96	27	17	140	43	14	199	2	258	932
	% HIV/AIDS	4.2	3.3	0	3.5	27.5	0	67.9	0	57.0	15.5
Kisesa	Deaths	97	127	141	365	68	224	90	11	393	914
	% HIV/AIDS	17.1	11.9	11.5	13.1	91.1	40.3	84.2	72.7	60.1	20.4
Manicaland	Deaths	60	60	84	204	–	639	–	–	639	186
	% HIV/AIDS	31.6	29.6	38.4	33.8	–	58.1	–	–	58.1	57.8
Masaka	Deaths	129	22	24	175	–	101	–	–	101	134
	% HIV/AIDS	27.0	21.0	7.8	23.6	–	57.5	–	–	57.5	34.6
Rakai	Deaths	78	77	67	222	507	230	921	76	1,734	1,424
	% HIV/AIDS	3.1	5.4	7.6	5.3	49.0	18.8	41.3	45.6	40.8	6.8
uMkhanyakude	Deaths	400	203	197	800	392	775	994	41	2,202	6,737
	% HIV/AIDS	1.7	2.9	6.2	3.1	35.8	21.3	36.7	32.2	31.0	15.0

The InterVA-4 model was ‘blinded’ to the HIV sero-status data, only taking into account a history or diagnosis of HIV/AIDS if reported in the VA interview.

**Table 2:** Specificities for InterVA-4 assignment of HIV/AIDS-related deaths, based on people with a negative HIV test result in the 5-year period before death

		Specificity	95% confidence interval
Overall		90.1	88.7–91.4
	15–49 years	83.3	80.6–85.6
Age group	50–64 years	92.7	89.5–94.9
	65+ years	97.4	95.9–98.4
	Male	94.0	92.3–95.3
Sex	Female	85.7	83.3–87.9
	1990–2002	77.2	71.4–82.1
Period	2002–2007	87.1	84.5–89.3
	2008–2011	95.8	94.3–96.9
	Karonga	96.5	92.0–98.5
	Kisesa	86.9	83.0–90.0
Study site	Manicaland	66.2	59.5–72.3
	Masaka	87.0	81.2–91.2
	Rakai	94.8	91.0–97.0
	uMkhanyakude	95.9	94.3–97.1

**Fig. 2:**
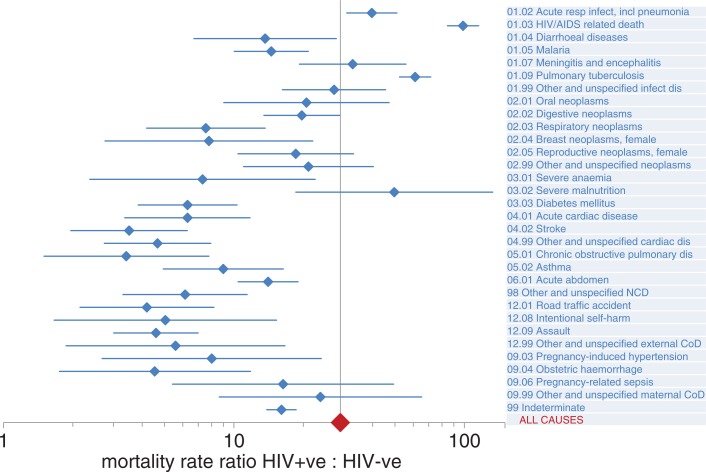
Cause-specific mortality rate ratios for HIV-positive: HIV-negative deaths, based on Poisson multivariate modelling of mortality rates, adjusted for age group, sex, and study site, showing 95% confidence intervals (CIs). The all-cause adjusted mortality rate ratio was 29.0 (95% CI 27.1–31.0), represented by the vertical axis.

For cases where HIV sero-status was known before death, it is possible to calculate cause-specific mortality fractions and rates according to HIV-negative and HIV-positive person-years observed. Table 3 shows results of this approach applied to 1,739 deaths in 1,161,688 HIV-negative person-years observed and 2,890 deaths in 75,110 HIV-positive person-years observed. The crude mortality ratio was thus 25.7, and on this basis 96% of the mortality in the HIV-positive group was excess over what would be expected in the absence of HIV. HIV-positive deaths attributed to HIV/AIDS, pneumonia, and tuberculosis accounted for most of this excess. For all causes, mortality rates were higher among the HIV-positive group, and for many the rate ratios were considerably greater than unity. A Poisson regression model was used to estimate cause-specific mortality ratios adjusted for age group, sex, and site. The all-cause adjusted mortality ratio was 29.0 (95% CI 27.1–31.0). Cause-specific adjusted mortality ratios, with 95% confidence intervals, are shown in Fig. 2. For all causes, HIV-positive people had significantly higher mortality than HIV-negative people (lower limits of 95% CIs for mortality ratios>1), but with considerable variation in ratios between causes, as shown on the horizontal logarithmic scale of Fig. 2.

**Table 3:** InterVA-4 causes of death, cause-specific mortality fractions, and adjusted mortality ratios by known HIV status for 1,739 deaths in 1,161,688 HIV-negative person-years observed and 2,890 deaths in 75,110 HIV-positive person-years observed

	Cause-specific mortality fraction (%)		Mortality rate per 1,000 per year		
		
WHO VA cause of death by InterVA-4	HIV negative	HIV positive	HIV negative	HIV positive	Mortality rate ratio
01.02 Acute respiratory infections, including pneumonia	4.16	6.33	0.06	2.44	39.1
01.03 HIV/AIDS-related death	10.60	38.89	0.16	14.96	94.3
01.04 Diarrhoeal diseases	1.07	0.36	0.02	0.14	8.6
01.05 Malaria	3.17	1.95	0.05	0.75	15.8
01.07 Meningitis and encephalitis	1.12	1.33	0.02	0.51	30.5
01.09 Pulmonary tuberculosis	11.54	28.71	0.17	11.05	63.9
01.99 Other and unspecified infectious disease	1.68	1.54	0.03	0.59	23.5
02.01 Oral neoplasms	0.49	0.37	0.01	0.14	19.2
02.02 Digestive neoplasms	3.92	1.98	0.06	0.76	13.0
02.03 Respiratory neoplasms	2.58	0.60	0.04	0.23	6.0
02.04 Breast neoplasms female	0.51	0.19	0.01	0.07	9.6
02.06 Reproductive neoplasms female	1.61	0.90	0.02	0.34	14.3
02.99 Other and unspecified neoplasms	1.85	0.56	0.03	0.21	7.7
03.01 Severe anaemia	0.80	0.11	0.01	0.04	3.7
03.02 Severe malnutrition	0.35	0.41	0.01	0.16	29.7
03.03 Diabetes mellitus	3.75	0.73	0.06	0.28	5.0
04.01 Acute cardiac disease	1.62	0.40	0.02	0.15	6.4
04.02 Stroke	4.73	0.40	0.07	0.15	2.2
04.99 Other and unspecified cardiac disease	5.31	0.69	0.08	0.27	3.4
05.01 Chronic obstructive pulmonary disease	2.82	0.17	0.04	0.07	1.6
05.02 Asthma	1.59	0.49	0.02	0.19	8.0
06.01 Acute abdomen	6.38	2.79	0.10	1.07	11.2
98 Other and unspecified non-communicable disease	2.45	0.44	0.04	0.17	4.6
12.01 Road traffic accident	2.71	0.47	0.04	0.18	4.4
12.08 Intentional self-harm	0.99	0.14	0.01	0.05	3.6
12.09 Assault	6.18	1.29	0.09	0.50	5.4
12.99 Other and unspecified external cause of death	1.57	0.23	0.02	0.09	3.8
09.03 Pregnancy-induced hypertension	0.38	0.14	0.01	0.05	9.1
09.04 Obstetric haemorrhage	1.09	0.15	0.02	0.06	3.6
09.06 Pregnancy-related sepsis	0.28	0.17	0.00	0.07	15.7
09.99 Other and unspecified maternal cause of death	0.37	0.33	0.01	0.13	22.9
99 Indeterminate	12.31	6.74	0.18	2.59	14.1
All causes	100	100	1.50	38.48	25.7

## Discussion

Overall, these results show that the InterVA-4 detects typical AIDS deaths in adults with high specificity, without the model having access to sero-status data. Exactly what constitutes a typical AIDS death is not easy to define, even though it is clear that the clinical expertise encapsulated within the InterVA model is, to a large extent, successfully identifying symptom patterns that are strongly associated with final illnesses in HIV-infected people, which might reasonably fall into the category of ‘HIV/AIDS-related death’. Nevertheless, HIV infection also clearly affects many other cause-specific mortality rates. Therefore, it is not possible to calculate a single parameter for sensitivity, because there is no single cause of death that inevitably follows HIV infection, and it is also evident that patterns of cause of death do vary considerably between the HIV-negative and HIV-positive groups, again without the model having access to those data.

Achieving 90% specificity overall in identifying typical HIV/AIDS-related deaths with the InterVA-4 model, although blinding the model to sero-status, is an important achievement. Interestingly, the number of false positives recorded only slightly exceeded the number of cases with a negative test during the previous 5 years where a diagnosis of HIV was subsequently declared in the VA interview. Because it is clear that HIV positivity is by no means universally disclosed in VA interviews (here not disclosed in 37.2% of the HIV-positive cases), it is likely that at least a proportion of the false positives identified here were in fact true positives by the time of death. The generally lower proportions of false positives who tested negative closer to their time of death also support this possibility. The recall period from death to VA interview varied by site, and this may have influenced specificity in some sites.

Although HIV infection represents a substantial risk factor for mortality from many causes, as shown in Fig. 2, it was also the case numerically, as shown in Table 3, that the overwhelming majority of deaths (73.9%) among HIV-infected people were assigned to acute respiratory infections, AIDS, or pulmonary TB. However, it is equally clear that not all of the 25-fold overall mortality risk among the HIV infected is accounted for by these main causes. As anti-retroviral therapy (ART) coverage increases in scope and effectiveness, the overall mortality risk associated with HIV infection is likely to decrease, but at the same time there is likely to be increasing diversity in cause of death among the HIV infected. Monitoring these changes in cause-specific mortality at the community level, using a standardised tool such as InterVA-4, will be an important strategy for understanding the future trajectory of the HIV pandemic.

Cause of death patterns for HIV-positive people in African populations are generally not well documented. An autopsy series from South Africa showed that the top three ranked causes of death among HIV-positive people were TB, pneumonia, and meningitis (20) and the same pattern was observed in a smaller series in Uganda (21). A review of autopsy studies in Africa showed that TB, pneumonia, and meningitis were major causes of death among HIV-positive people, though there were major discrepancies between hospital causes of death and pathology findings (22). Similar causes of death patterns have been observed in hospital patient series in Thailand (23) and South Africa (24). Comparisons between various studies are sometimes difficult to make because of a variety of understandings of what ‘AIDS deaths’ constitute against a background of specific causes of death such as pneumonia and meningitis among the HIV positive. The very different nature of HIV epidemiology in other settings, such as Taiwan (25) and Korea (26), also make comparison with African populations difficult. However, the VA findings from this study are broadly consistent with these other cause of death findings among HIV-positive people.

Lopman et al. proposed a number of key symptoms that were associated with HIV infection (27), many of which are directly reflected in the causes of death with high rate ratios associated with HIV positivity here. The largest numbers of relevant symptoms involved relate to pulmonary TB and acute respiratory infections, although there are very high rate ratios for symptoms associated with some rarer causes as well. An indirect comparison can also be made with excess risks among transplant patients who are artificially immunosuppressed to counter-rejection. A population-based study in Sweden showed very high incidence rates for some specific cancers among immunosuppressed transplant patients: lip [standardised incidence ratio (SIR) 53]; mediastinum (SIR 43); vulva and vagina (SIR 21); and non-melanoma skin cancer (SIR 56) (28). Although VA does not readily enable highly detailed site-specific identification of neoplasms, the very high mortality rate ratios for oral and female reproductive neoplasms in Table 3 are not inconsistent with these findings from Sweden.

As might be expected in a multi-site study, there were some differences observed between sites, and these are likely to be due to a combination of procedural and methodological differences, as well epidemiological and geographical variation. In many ways recovering VA data retrospectively from a variety of instruments is a worst-case scenario, even if a realistic one; at least it is very unlikely to have enhanced the performance of cause of death assignment. We would expect that VA interviews conducted directly under the WHO 2012 standard protocol would yield higher quality data. A further important factor that will have changed over time, but is not documented here, is the uptake of ART. As yet it remains an open question as to what the cause of death patterns may turn out to be among HIV-positive people who have taken ART for many years or even decades. It already appears here that there was a lower proportion of typical AIDS deaths among all categories of HIV positivity in the later periods, and this may well be due, at least in part, to treatment effects. The WHO 2012 VA standard (and hence InterVA-4) does not include any specific item on ART, though this may be something that needs to be incorporated in a future version.

Despite the difficulties in making precise comparisons of causes of death, the major differences between cause-specific adjusted mortality rate ratios by HIV status generated by InterVA-4 are important, given the blinding of the model to HIV sero-status. Unlike the relatively small post-mortem series that have been reported, this study of nearly 5,000 VAs on deaths of known HIV status allows examination of relatively rare causes of death in relation to HIV, as well as confirming the major causes of death related to the risk of HIV infection. Although VA as an overall approach, and the InterVA-4 model as a specific tool for determining cause of death, cannot be regarded as equivalent to undertaking post-mortems, this study shows that VA can realistically be applied in large-scale community-based settings and yield cause of death results that are consistent with other approaches, successfully differentiating by HIV status. In addition, the use of a standardised model such as InterVA-4 for interpreting VA material obviates the possibility of local differences in interpretation. We conclude that InterVA-4 is an effective and valid public health tool for assessing mortality in relation to HIV infection and deaths due to AIDS.
